# Complete Genome Sequence of *Bordetella bronchiseptica* S798, an Isolate from a Pig with Atrophic Rhinitis

**DOI:** 10.1128/genomeA.00436-14

**Published:** 2014-05-15

**Authors:** Keisuke Okada, Yoshitoshi Ogura, Tetsuya Hayashi, Akio Abe, Asaomi Kuwae, Yasuhiko Horiguchi, Hiroyuki Abe

**Affiliations:** a Department of Molecular Bacteriology, Research Institute for Microbial Diseases, Osaka University, Osaka, Japan; b Division of Microbial Genomics, Department of Genomics and Bioenvironmental Science, Frontier Science Research Centre, University of Miyazaki, Miyazaki, Japan; c Division of Microbiology, Department of Infectious Diseases, Faculty of Medicine, University of Miyazaki, Miyazaki, Japan; d Laboratory of Bacterial Infection, Graduate School of Infection Control Sciences, Kitasato University, Tokyo, Japan

## Abstract

*Bordetella bronchiseptica* colonizes the respiratory tracts of a wide variety of mammals and causes a range of diseases, from lethal pneumonia to asymptomatic chronic infection. We report the complete genome sequence of *Bordetella bronchiseptica* strain S798, isolated from a pig with atrophic rhinitis in Japan.

## GENOME ANNOUNCEMENT

*Bordetella bronchiseptica*, a Gram-negative bacterium, is considered to have an evolutionary progenitor-like relationship to *Bordetella pertussis* and *Bordetella parapertussis*, which are causative agents of pertussis in humans (1). *B. bronchiseptica* is known to cause respiratory infections such as atrophic rhinitis in pigs, snuffles in rabbits, kennel cough in dogs, and occasionally asymptomatic chronic infections in various mammals (2). Multilocus sequence typing and comparative genomic hybridization analysis of *B. bronchiseptica* strains isolated from different hosts revealed that genetic differences may be associated with adaptation to the different hosts and different manifestations of disease (3, 4). The genomes of *B. bronchiseptica* strains isolated from a rabbit, a dog, and humans have been sequenced. To elucidate the underlying genetic characteristics associated with atrophic rhinitis in pigs, we determined the complete genome sequence of *B. bronchiseptica* strain S798, isolated from a pig with atrophic rhinitis in Japan.

The genome sequence of S798 was determined by a combined strategy of 454 GS FLX Titanium pyrosequencing (Roche) and Sanger sequencing. A total of 512,672 single-end reads (209 Mb) and 304,307 8-kb paired-end reads (51 Mb) were assembled with GS Assembler software version 2.6 into one scaffold containing 133 gaps. Gap closing and resequencing of low-quality regions were performed by sequencing the PCR products. The genome sequence was automatically annotated with the Microbial Genome Annotation Pipeline (MiGAP) (http:/www.migap.org/). The start codon positions and additional genes that were missing in the MiGAP annotation were manually inspected and corrected. The complete genome of S798 contains a circular 5,191,712-bp chromosome with an average G+C content of 68.21%. There are 4,791 DNA coding sequences (CDSs), with an average product size of 327 amino acids, and 54 tRNA genes and 3 rRNA operons in the genome. The genome of S798 was found to carry two large fragments (>10 kb) including 8 and 13 CDSs and 28 fragments (<10 kb) including 67 CDSs which were absent in strain RB50, isolated from a rabbit. Among them, 18 CDSs were exclusively present in S798, being absent in the strains 253 and MO149, whose genomes were already sequenced (1, 6). Twelve S798-specific CDSs were annotated as encoding hypothetical proteins with unknown functions.

The *dnt* gene, encoding dermonecrotic toxin, which is a virulence factor causing turbinate atrophy in swine atrophic rhinitis (5), was highly conserved among S798, RB50, and 253, isolated from nonhuman hosts (over 99% identity). Other virulence factors such as filamentous hemagglutinin, pertactin, fimbriae, adenylate cyclase toxin, and the type III and type VI secretion systems were also conserved among them, whereas a gene encoding *Bordetella* colonization factor A (BcfA) in S798 was more similar to the *bcfA* genes in MO149, isolated from a human, and *B. parapertussis* 12822 than to that in RB50 (7). A detailed comparison of the genome sequence of S798 with those of other *B. bronchiseptica* strains might provide some insights into the pathogenesis specific to pigs.

### Nucleotide sequence accession number.

The complete genome sequence of *B. bronchiseptica* S798 has been deposited in the DDBJ/EMBL/GenBank database under accession number AP014582.
